# Macrophages in gastrointestinal homeostasis and inflammation

**DOI:** 10.1007/s00424-017-1958-2

**Published:** 2017-03-10

**Authors:** John R. Grainger, Joanne E. Konkel, Tamsin Zangerle-Murray, Tovah N. Shaw

**Affiliations:** 1 Manchester Collaborative Centre for Inflammation Research (MCCIR), University of Manchester, Manchester, M13 9NT UK; 2 Faculty of Biological, Medical and Human Sciences (FBMH), University of Manchester, Manchester, UK

**Keywords:** Macrophage, Monocyte, Mucosal, Gastrointestinal, Commensal, Inflammatory bowel disease

## Abstract

Monocyte-derived mononuclear phagocytes, particularly macrophages, are crucial to maintain gastrointestinal homeostasis in the steady state but are also important for protection against certain pathogens. However, when uncontrolled, they can promote immunopathology. Broadly two subsets of macrophages can be considered to perform the vast array of functions to complete these complex tasks: resident macrophages that dominate in the healthy gut and inflammation-elicited (inflammatory) macrophages that derive from circulating monocytes infiltrating inflamed tissue. Here, we discuss the features of resident and inflammatory intestinal macrophages, complexities in identifying and defining these populations and the mechanisms involved in their differentiation. In particular, focus will be placed on describing their unique ontogeny as well as local gastrointestinal signals that instruct specialisation of resident macrophages in healthy tissue. We then explore the very different roles of inflammatory macrophages and describe new data suggesting that they may be educated not only by the gut microenvironment but also by signals they receive during development in the bone marrow. Given the high degree of plasticity of gut macrophages and their multifaceted roles in both healthy and inflamed tissue, understanding the mechanisms controlling their differentiation could inform development of improved therapies for inflammatory diseases such as inflammatory bowel disease (IBD).

## Introduction

The mammalian intestine is a complex environment for the immune system. On one hand, it must maintain tolerance to a vast array of antigens derived from food and the dense, but largely harmless, commensal microbiota. On the other, it must be ever ready to respond to potentially life-threatening pathogens that aim to colonise via the oral route. Failure to achieve this knife-edge balance between tolerance and responsiveness can lead to mortality or life-limiting morbidity, as occurs in inflammatory bowel disease (IBD).

For ongoing homeostasis to be achieved in the gut, interrelated highly specialised structural and cellular strategies have, thus, evolved to support this immunologic balancing act. All the time allowing the tissue to perform its primary physiologic function—absorption of nutrients, water and electrolytes. These structures include a mucus layer that creates a physical barrier to keep bacteria away from the epithelium, a single-cell thick epithelial layer and a specialised immune network enriched in the epithelial layer and *lamina propria*.

Resulting from their phagocytic capacity, functional plasticity and capability to integrate and interpret diverse food-derived, commensal-derived, pathogen-derived and host-derived signals in their environment, gut-*resident* macrophages are well established as a keystone immune population in barrier homeostasis in health. Moreover, following initiation of an inflammatory response, *inflammation-elicited (inflammatory)* macrophages (derived from circulating blood monocytes called into the affected tissue) in tandem with their resident partners play crucial roles in control of infection. Thus, in the face of the myriad challenges that the intestine will face over a lifetime, the dynamic regulation of the intestinal macrophage pool is at the centre of long-term health.

Macrophage biology is a field that has seen explosive growth in recent years, particularly in the gut. A large number of studies, including our work, have begun to establish how the ontogeny and differentiation of these cells is tailored by, and to, the gut environment. In this article, we will discuss these findings and particularly explore the unique mechanisms governing resident and recruited inflammatory gut macrophage function.

### Location and functions of resident macrophages in the healthy gut

**Fig. 1:**
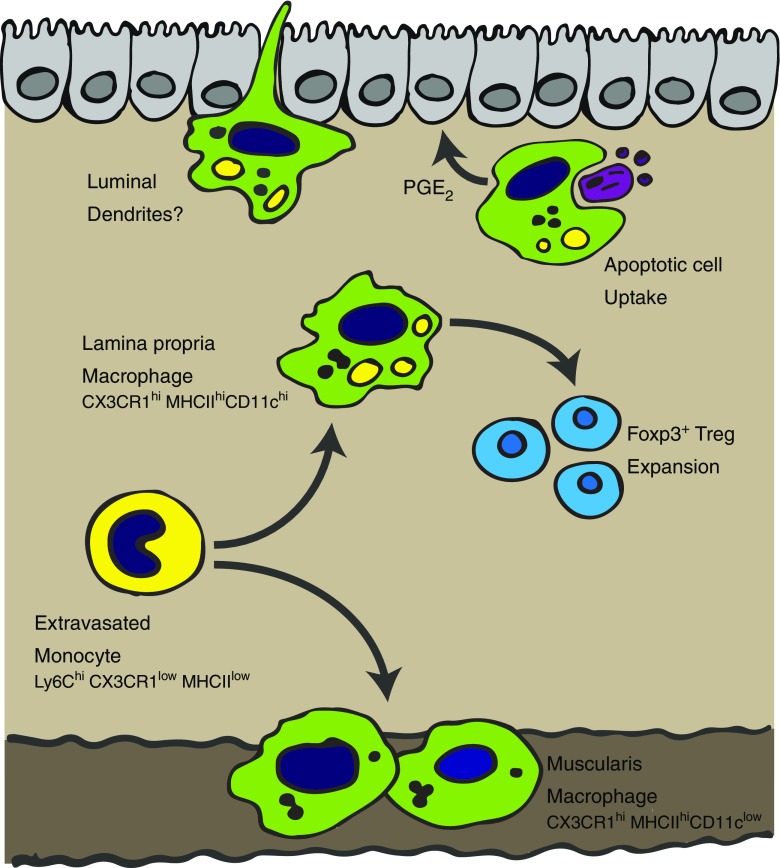
Development and functions of resident intestinal macrophages. In the healthy gut, Ly6C^hi^ monocytes are constantly recruited from the blood into the gut to replenish the resident macrophage pool. Ly6C^hi^ monocytes transit through a series of phenotypically defined stages to eventually become mature CX3CR1^hi^MHCII^hi^Ly6C^low^ macrophages in the *lamina propria* and muscularis. These macrophages are hyporesponsive to bacterial ligands, constitutively produce IL-10 and have multiple crucial functions in gut homeostasis including Treg expansion, epithelial maintenance, luminal sampling and bacterial killing

The largest population of resident macrophages in the body is present in the steady-state intestine [42, 56]. They are found along the entire length of the intestine, from the proximal small intestine to the distal large intestine, and are enriched in the *lamina propria* (LP) close to the epithelial layer (see Fig. 1) [42]. There is also a morphologically distinct population present in the smooth muscle layers [21]. Along the length of the gut, the number of macrophages varies, with the highest density found in the colon of both humans and rodents [16, 70].

As in most tissues, a key role of macrophages in the gut is to perform housekeeping functions such as tissue remodelling and removal of senescent or dying cells [14, 70, 78]. In line with this function, they have high expression of scavenger receptors, including CD36, that are able to support apoptotic cell uptake [95]. They are also able to produce soluble factors that can help to support epithelial barrier integrity such as the lipid mediator prostaglandin E_2_ (PGE_2_) [25]. Additionally, macrophages located in the muscularis and serosa are important in interacting with nerves to support peristalsis, ensuring ongoing movement of ingested material along the intestine [69].

However, alongside these homeostatic functions, macrophages in the gut are also important immune sentinels and effector populations. The positioning of LP macrophages in close apposition to the epithelial layer means that they are able to rapidly uptake and respond to any material breaching this barrier [42]. These resident macrophages are highly phagocytic and have bactericidal properties [96]. They can also produce chemokines to recruit effector cells from the blood into the tissue when required [95]. Intriguingly, unlike other studied macrophage populations in the body, although gut macrophages can respond to bacteria, they do not induce classic pro-inflammatory responses [4, 96]. This is a critical feature to prevent aberrant inflammation towards the high commensal bacterial load [118]. For example, ingestion of bacteria does not lead to enhanced respiratory burst activity [80], and ligation of toll-like receptors (TLRs) or nucleotide-binding oligomerisation domain (NOD)-like receptors (NLRs) does not result in increased TNF-α or IL-6 production [34, 96]. Despite this hyporesponsive phenotype towards bacteria, intestinal macrophages are not completely unresponsive in their cytokine-producing capacity, and they constitutively produce the anti-inflammatory cytokine IL-10 and low levels of TNF-α [4]. Although this production of TNF-α may at first seem counterintuitive (due to its inflammatory capacity), TNF-α can impact enterocyte growth [61] as well as modulating production of matrix metalloproteinases from intestinal mesechymal cells [75], actions which are suggested to allow gut macrophages to support the maintenance of barrier homeostasis [3].

An important component of the gut immune system that is critical in establishing tolerance towards the high burden of food and commensal antigens is the forkhead box protein 3 (Foxp3)^+^ T regulatory cell (Treg) network [65]. Since gut macrophages are able to uptake orally acquired antigens and also express high levels of major histocompatibility complex class II (MHCII) [4, 65], it is not surprising that they are suggested to play a key role in supporting development of this network. Of note, Hadis et al. demonstrated that following their priming in the lymph node, antigen-specific Foxp3^+^ Tregs that had trafficked to the LP were maintained at this site by macrophages [32]. A similar role for macrophages in contributing to the tissue-resident Treg pool has also been suggested in the lung [98].

It is possible that gut macrophages may play roles in supporting maintenance of other T cell subsets in the gut. In particular, macrophages can produce IL-1β following TLR stimulation, and this has been suggested to support Th17 cell development in the healthy gut [93]. Along these lines, recruited macrophages have also been shown to support generation of commensal-specific Th17 cells [74]. Although gastrointestinal-resident Th17 cells are key mediators of barrier defence [113], dysregulated Th17 responses are a driver of colitogenic pathology, and in settings of gastrointestinal inflammation, macrophages have been shown to support amplification of Th17 responses [58]. Further studies will be required to establish whether macrophage education of gastrointestinal T cell populations requires cognate MHCII-T cell receptor interactions or whether the cytokine milieu established by macrophages is the major factor. Moreover, the influence of macrophages on other gastrointestinal-resident lymphocyte populations during steady state requires further assessment, especially in the light of reports detailing macrophage-innate lymphoid cell (ILC) interactions which are key to support gastrointestinal immune homeostasis and reinforce barrier integrity [63, 68, 88].

Another way in which gut macrophages may be able to support development of the gut T cell network in an indirect manner is by transfer of soluble antigen from the lumen to gut dendritic cells (DCs) [65]. These DCs then drain to the mesenteric lymph nodes to prime T cell responses [85]. It is still not entirely clear how LP macrophages acquire these luminal antigens, but one possibility is that they can extend transepithelial dendrites across the epithelium [10, 72]. However, this idea remains controversial with original reports disagreeing on which parts of the small intestine these transepithelial dendrites were present in and the necessity for TLR signalling, and subsequent studies unable to identify them [10, 51, 72].

An issue that has confounded functional studies of gut macrophages has been the complexities of identifying this population using flow cytometry [3, 8, 41]. Initially, this problem largely arose due to the assumption that CD11c and MHCII were markers of gut DCs. In the gut, LP macrophages express high levels of CD11c as well as MHCII and thus in many studies were assumed to be DCs [6, 10]. Unlike macrophages, DC constantly drain from tissue to lymph nodes where their major role is to prime naïve T cells [85]. More recently, the fractalkine receptor CX3CR1 has been used to distinguish gut macrophages from gut DC [6, 85, 111]. Notably, resident gut macrophages express high levels of CX3CR1; however, some subsets of gut DC also express this marker (albeit at an intermediate level), which may be the basis of more recent contradictory findings regarding DC and macrophage function [3, 8, 41].

One problem with using high expression of CX3CR1 as a marker of gut macrophages is that this can currently only be established using CX3CR1-GFP transgenic mice and not by antibody staining [46]. A more useful strategy for identifying gut macrophages by multicolour flow cytometry (that can be used in non CX3CR1-GFP expressing animals) came from large-scale genomic datasets alongside more targeted studies [2, 4, 23, 104]. Together, these publications have identified that CD64 (FcγRI), F4/80 and MHCII used in combination with Ly6C and lineage exclusion markers (e.g. for lymphocytes and granulocytes) can reliably define these cells. CD11c expression was found to be of particular relevance to distinguish LP macrophages (CD11c^hi^) from those in the serosa and muscularis (CD11c^low^) [6]. When isolated by fluorescence-activated cell sorting (FACS), these cells exhibit characteristic macrophage morphology and cannot be found draining to the mesenteric lymph nodes [2, 41]. Moreover, their development is critically dependent on colony stimulating factor 1 receptor (CSF1R) (also known as macrophage colony-stimulating factor receptor (MCSFR)) [82, 83] but independent of the DC growth factor FMS-like tyrosine kinase 3 ligand (FLT3L) [104]. Altogether, these findings suggest that CD64 and F4/80 are bona fide markers of gut-resident macrophages and will support researchers in specifically determining gut macrophage functions in future studies.

### The unusual ontogeny of resident gut macrophages

When the mononuclear phagocyte system (MPS) was first described just over 50 years ago, it was proposed that tissue macrophages were the terminal differentiation stage of blood monocytes after recruitment into tissue [110]. However, in more recent years, a number of studies have been published that demonstrate that the majority of tissue macrophages are able to exist independently of blood monocyte precursors [33, 38, 39]. Frequently these derive from foetal liver precursors although some, including the microglia of the central nervous system (CNS), come from the yolk sac [24, 38, 39, 116]. These cells seed tissues prenatally and then are maintained by local proliferation. Resident macrophages include those of the lung alveoli [116], the Kuppfer cells of the liver [87] and epidermal Langerhans cells [39].

The adult intestinal macrophage pool is a major exception to this rule (along with the dermis [103] and more controversially the heart [18, 66]) fitting the original MPS model, requiring constant replenishment from blood monocytes (see Fig. 1) [2, 4, 6, 119]. Blood monocytes are a heterogeneous circulating population in both humans and mice that originate in the bone marrow (BM). In mice, there is a subset that expresses high levels of Ly6C and CCR2 termed “classical” monocytes (the equivalent of human CD14^hi^ monocytes [43]) that are the precursors to the adult intestinal macrophages [2, 4, 6, 119]. Although at birth, there are embryonically derived macrophages present in the gut, around the time of weaning these are replaced by cells derived from an influx of CCR2-dependent Ly6C^hi^ monocytes [2].

A number of studies investigating macrophage differentiation in the healthy gut have been instrumental in defining the phenotypic and transcriptional profile of classical (Ly6C^hi^) blood monocytes transitioning into mature gut macrophages. Initially using an adoptive transfer approach in healthy gut, it was shown that Ly6C^hi^ monocytes were able to enter into the colon and mature into CD64^+^F4/80^hi^CX3CR1^hi^MHCII^+^ macrophages [4]. This developmental process involves a series of identifiable intermediaries in which Ly6C expression is lost while expression of F4/80, CX3CR1, CD163 and CD206 are gained. Due to the visual appearance of this transition moving from Ly6C^hi^ to MHCII^hi^ or CX3CR1^hi^ on a flow cytometry plot, this has been referred to as the monocyte to macrophage “waterfall” [2, 104]. Differentiation takes approximately 5 days and results in a cell that has increased phagocytic capacity and constitutive IL-10 production and is anergic to TLR stimulation. This rapid differentiation is in line with earlier reports of an approximately 3–5-week half-life for gut-resident macrophages [45]. Although the precise mechanisms governing differentiation cannot be easily established in humans, resident gut macrophages deriving from blood monocytes is implied by a similar waterfall of CD14^hi^ (marker characteristic of classical monocytes [43]) to CD14^low^CD209^hi^CD163^hi^ cells [4].

The complex commensal flora is a distinguishing feature of the intestine, and there is a current consensus that it is this feature that is important in regulating the continuous replenishment of resident gut macrophages from blood monocytes [2, 3, 118]. Of note, there is a first accumulation of colonic macrophages between 2 and 3 weeks of age in mice that is concurrent with increased commensal colonisation [2]. Corroborating the importance of the microbiome in this process, at 3 weeks of age, there were fewer mature macrophages in germ-free mice than conventionally housed controls. Moreover, many of these macrophages did not express MHCII further implicating the microbiome in typical differentiation of macrophages as well as recruitment [4, 119].

It is worth noting, however, that this was not the first study to investigate colonic macrophage abundancy in germ-free animals, and these studies have reached opposing conclusions [71, 79, 108]. Animals from different sources are now well known to have very different commensal composition [11, 44, 107]. One hypothesis for these differences between studies is that there are unique factors in the gut other than the microbiome that can affect macrophage replacement from blood monocytes but that specific bacterial species present in certain mouse colonies can enhance or decrease the turnover rate.

Taken together, the studies to date strongly suggest that in both mice and humans, and in stark contrast to other tissues, resident gut macrophages are continuously replenished from circulating blood monocytes. Whether this information can be used to design strategies to specifically target gut macrophages remains to be explored but suggests that they may be impacted by systemic drug administration in a way that macrophages maintained locally in tissue would not.

### Instruction of resident gut macrophage function

The main cues present in the gut environment that are responsible for monocyte to resident macrophage differentiation are still poorly defined. However, it is likely that there are specific signals or combinations of signals in the gut that induce characteristics not observed at other mucosal sites such as the skin and lung [55, 86, 103, 109].

There are a number of important growth factors involved in the establishment of the MPS, notably, FLT3L, CSF1 (MCSF) and CSF2 (granulocyte macrophage colony-stimulating factor). Although FLT3L and CSF2 may have impacts on gut macrophage function [68], they appear to be dispensable for their development [26, 104]. This is in stark contrast to lung macrophages for which CSF2 signalling is extremely important [30, 101]. Two ligands have been identified for CSF1R: CSF1 itself and IL-34 [112]. It seems most likely that development and maintenance of macrophages in the gut are CSF1 dependent as there is a marked reduction of macrophages in mice that have a mutation in the gene that encodes CSF1 (*Csf1* ^*op/op*^) or following administration of anti-CSF1R antibody [69, 82].

One particularly unusual phenotype of resident gut macrophages is their acquisition of high levels of CX3CR1 expression. In the majority of tissues, CX3CR1 is downregulated on macrophages suggesting that there is a factor in the gut responsible for its continued expression. A recent study has suggested that TGF-β, which is established to induce CX3CR1 expression in the brain [9], is also a dominant signal for CX3CR1 expression by gut macrophages [84]. This is in line with the identification of Runx3 as a characteristic transcription factor of gut macrophages that is regulated by TGF-β in T cells [52, 53, 55]. In addition, it is possible that the interaction of CX3CR1 with CX3CL1 (fractalkine) may be important itself in instructing gut macrophage function. For example, it has been suggested that CX3CR1-CX3CL1 interactions are critical for optimal production of IL-10 by macrophages [32].

IL-10 signalling is one pathway that has been shown to be crucial for instructing macrophage function in the gastrointestinal (GI) mucosa [94, 117]. Not only do macrophages make IL-10, but IL-10 signals to the macrophage are vital for the appropriate education of intestinal macrophage populations, providing a key cue that safeguards their hyporesponsive phenotype (discussed below). It has long been known that in the absence of IL-10, severe gastrointestinal inflammation results [54]; what is now clear is that IL-10 receptor signalling on macrophages, but not the production of IL-10 itself, is key in restraining this inflammation [94, 117]. When unable to respond to IL-10, gut macrophages produce increased levels of inflammatory cytokines in response to bacterial stimulation [102, 108] and drive development of colitis [40]. More recently, specific deletion of IL-10Rα on CX3CR1-expressing gut macrophages was shown to lead to the development of a spontaneous, and severe, ulcerative colitis-like GI inflammation [117]. Part of this IL-10 conditioning of macrophage function has been reported to include limiting levels of NOS2, PGE_2_, IL-23 [117], inflammasome components and pro-IL-1β [19, 31, 58]. Thus, IL-10 signals have emerged as a vital restraint against overt macrophage activation. Interestingly, this pathway has also been implicated in early onset IBD, as similar functional alterations to those seen in animal models have been observed in patients with IL-10R mutations [94].

As mentioned, another key feature of gut macrophages is their apparent hyporesponsiveness to TLR-mediated activation. Intriguingly, gut-resident macrophages both in humans and mice express a full repertoire of TLR receptors, so it is thought that it is downstream mediators that are responsible for the hyporesponsiveness [4, 97]. Of note, molecules including MyD88, TRAF-6, TRIF and CD14 are downregulated in mature macrophages [96, 97, 119]. Additionally, there may be alternative pathways that impair TLR responsiveness, for example, through increased expression of IRAK-M and IκΒNS [37, 97, 119].

An emerging mediator of gut macrophage function is neuroendocrine signals, controlling macrophage survival and phenotype within the highly innervated gastrointestinal mucosa. Neuronal-macrophage interactions are bidirectional with macrophages in the muscularis capable of controlling peristaltic activity. Bone morphogenetic protein 2 (BMP2), produced by macrophages, acts on enteric neurons to control smooth muscle contractions and thus, peristalsis [69]. In return, neuroendocrine signals to macrophages support maintenance of macrophage populations within specific gastrointestinal niches. Bogunovic and colleagues demonstrated that enteric neurons ensure maintenance of macrophages specifically within the muscularis layer of the gastrointestinal tract through production of CSF1 [69] (as discussed a key growth factor in gut macrophage development). This neuroendocrine control of macrophage survival was downstream of commensal bacteria colonisation as microbial-derived signals promoted enteric neuron expression of CSF1. Additional macrophage niche specialisation by neuroendocrine pathways can also be driven by norepinephrine signals from sympathetic neurons. Muscarlis macrophages express β2 adrenergic receptors that, upon binding of norepinephrine released from local active neurons, promote acquisition of a tissue-protective programme in muscularis-resident macrophages during infection [21].

Intriguingly, and in line with microbial activation of CSF1 production from enteric neurones [69], it has also been reported that the microbiota controls glial cell homeostasis in the intestine [47]. These supporting cells provide key maintenance and protection for neurons further highlighting the complex integration of microbial, endocrine and immune signals in controlling GI immune homeostasis and inflammation. The very recent focus on neuroendocrine signals in control of immune cell function within the gut [64] will likely yield further insights into tissue training of macrophage function by these signals.

In addition to the signals described, there are many other factors expressed in the gut environment that may play important roles on macrophages but as yet have been poorly explored. These factors include thymic stromal lymphopoietin (TSLP), the mucus layer itself that has been shown to modify DC function [92] and retinoic acid. Retinoic acid is particularly intriguing as it is known to have profound effects on DC in the gut and in tandem with TGF-β support induction of Treg [12, 100]. The impact of RA on macrophages has yet to be precisely elucidated.

### Inflammatory gut macrophages in experimental settings of classical inflammation

**Fig. 2:**
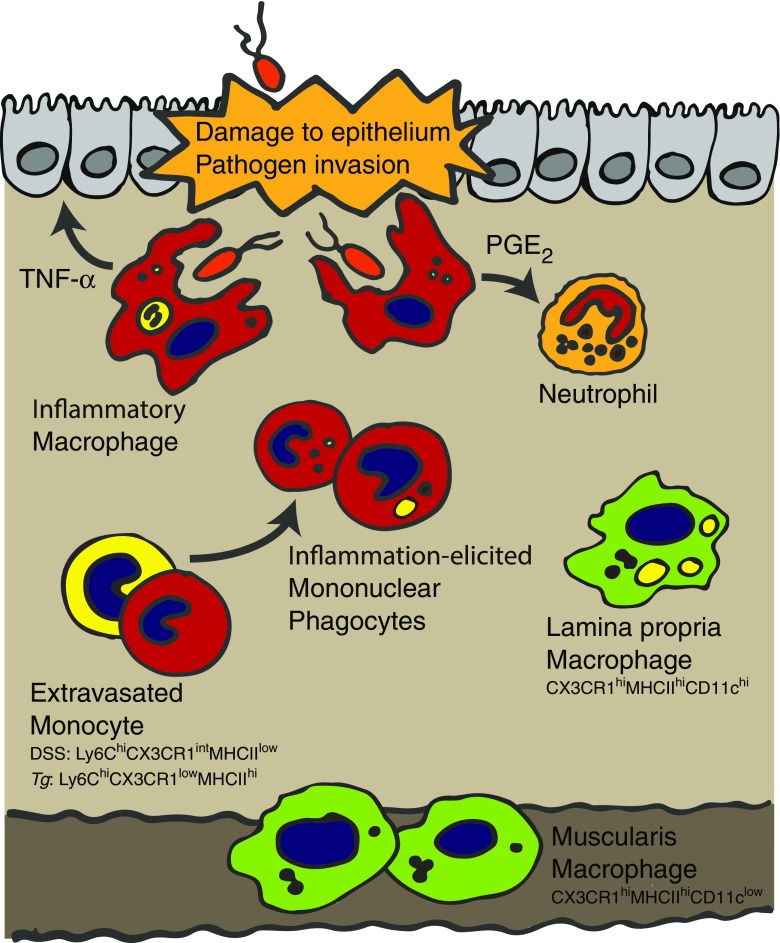
Macrophages during gut inflammation. Following epithelial damage or pathogen invasion, classical monocyte-derived effector cells are elicited and enter the now inflamed GI tract. The functions of these populations are poorly defined but likely highly specialised to the precise challenge and may include DC-like activities. Many differentiate into macrophages that are crucial for pathogen control but can also lead to pathology as a result of their potential to produce inflammatory cytokines such as TNF-α. During *T. gondii* infection, these cells also take on the capacity to suppress neutrophil activation via release of PGE_2_. Of note, during *T. gondii* infection, instruction of macrophage function begins in the bone marrow resulting in monocytes entering the tissue in a primed state characterised by their low expression of CX3CR1 and high expression of MHCII. Although monocytes no longer differentiate to resident macrophages (CX3CR1^hi^ *lamina propria* and muscularis macrophages) during inflammation, those present prior to barrier breach remain in the tissue and continue to exhibit anti-inflammatory features

Infiltration of classical (Ly6C^hi^) monocytes that differentiate rapidly into effector cells is a common feature following intestinal damage and infection of mice [4, 25, 104, 119] (see Fig. 2). Perhaps the best-studied murine models of this are colitis induced by administration of dextran sodium sulphate (DSS) and T cell transfer colitis [4, 104, 119]. In both of these settings, there is a characteristic reversal in the composition of the macrophage compartment. In particular, CX3CR1^hi^-resident gut macrophages that dominate in the healthy gut are outcompeted by inflammation-elicited CX3CR1^int^ mononuclear phagocytes (MNPs) that are the progeny of rapidly infiltrating Ly6C^hi^ monocytes [4, 119]. Precise definitions of the functions of this MNP pool are complex, and the cells within this pool are on a differentiation spectrum that likely includes populations with more macrophage-like or DC-like functions [119]. For the rest of this review, focus will be placed on the macrophage-like features of these cells that are present in inflammation that for clarity will be referred to as inflammatory macrophages (although the reader should note, and as will be discussed, that their functional and differentiation potential as well as morphology is very different to resident gut macrophages). These cells have a strong pro-inflammatory signature characterised by the production of factors including TNF-α, IL-1β, IL-6 and inducible nitric oxide synthase (iNOS). Of note, despite their presence in this pro-inflammatory milieu, the resident CX3CR1^hi^ macrophages continue to maintain the largely anti-inflammatory characteristics they exhibit in health [4, 104, 114, 119]. Not surprisingly, given their pro-inflammatory features, there is strong evidence for a pathologic role for the CX3CR1^int^ macrophages in these murine models of colitis. For example, in *Ccr2* ^−/−^ mice (which have a paucity of monocyte-derived cells in circulation and tissues due to a failure in classical monocyte release from the bone marrow [89]), or animals depleted of CCR2-expressing cells [119], there is an amelioration of DSS-driven colitic inflammation.

One intriguing feature of the Ly6C^hi^ monocyte differentiation pathway to inflammatory macrophages during colitis is the apparent concurrent impairment in Ly6C^hi^ monocyte differentiation to resident CX3CR1^hi^ macrophages [4, 104, 119]. Indeed, it has been proposed that this arrest in the differentiation pathway may underlie the ongoing capacity of inflammatory macrophages to respond to TLR stimulation in DSS colitis. The precise mechanisms that result in such changes are currently unknown but are likely due to signals being altered in the local microenvironment that are critical for educating monocytes to become resident anti-inflammatory macrophages.

Despite inflammatory macrophages playing immunopathogenic roles during acute colitic-like inflammation, in certain gastrointestinal infections, they are crucial for pathogen protection [17, 25]. Following oral inoculation with the highly type 1 polarising intracellular protozoan parasite *Toxoplasma gondii*, there is dramatic infiltration of Ly6C^hi^ monocytes into the inflamed small intestine [17, 25]. Once again, the importance of monocytes in this setting can be revealed by the fact that CCR2-deficient mice or those deficient in its ligand CCL2 fail to control the parasite [17, 25], and this can be restored by monocyte transfer [17].

Although, this parasite control was initially attributed to the capacity of recently recruited macrophages to produce pro-inflammatory cytokines such as TNF-α and iNOS, their function has since been revealed to be more complex [25]. Our studies demonstrated that in CCR2-deficient animals infected with *T. gondii*, neutrophils became hyperactivated in the *lamina propria* of the gut where they produced dramatically increased levels of tissue damaging factors including TNF-α and reactive oxygen species (ROS) [25]. This was associated with increased severity of gastrointestinal pathology that was independent of increases in parasite load. Immunofluorescent imaging revealed that Ly6C^hi^ inflammatory macrophages localised closely with neutrophils in the inflamed gut suggesting that one explanation for their enhanced activity might be direct suppressive actions of Ly6C^hi^ macrophages. Supporting this idea, we found that treatment of neutrophils with factors released from Ly6C^hi^ macrophages isolated from *T. gondii*-infected guts limited the neutrophils’ capacity to produce pro-inflammatory factors in response to TLR ligands and formyl peptides. Further experiments revealed that this effect was entirely dependent on the release of the lipid mediator PGE_2_, which is highly expressed by inflammatory macrophages during *T. gondii* infection [1, 25] (see Fig. 2). This lipid mediator can have complex and opposing roles over the course of an inflammatory response as it can favour inflammatory cell recruitment [48] but is also a potent suppressor of human neutrophil activation [115].

Following its initial invasion of the gut, *T. gondii* infection eventually disseminates systemically and inflammatory macrophages infiltrate lymphoid tissues including the spleen [25]. However, the neutrophil-suppressive activity was only evident in macrophages isolated from the gut. Studies using germ-free (GF) mice revealed that this was critically dependent on commensal-derived ligands. The precise bacteria involved in instructing macrophage function during inflammation are likely very different to those resident macrophages are exposed to in health. Notably, in the context of gut infection, there is tremendous outgrowth of potentially pathogenic commensal populations, specifically γ-proteobacteria such as *Escherichia coli* [35, 67]. Outgrowth of γ-proteobacteria has also been reported in patients with IBD [15, 59]. Indeed, in vitro treatment of circulating Ly6C^hi^ monocytes from the blood of *T. gondii*-infected animals with a lysate from a commensal form of *E. coli* led to rapid PGE_2_ production. Of course, in vivo, there may be additional gut-specific signals responsible for initiating PGE_2_ release. For example, stimulated epithelial cells can produce and activate IL-1β [99], a strong driver of PGE_2_ from macrophages [76], and commensal-derived dietary ligands such as short chain fatty acids (SCFAs) can also augment PGE_2_ production [13]. It is interesting to speculate that in certain individuals or at defined time points during infection, alterations in the composition of commensal species may be able to favour acquisition of regulatory features by infiltrating monocytes.

Another mechanism by which inflammatory macrophages may act to suppress immune cells during inflammation is via actions on T cells. This could be achieved by modifying L-arginine metabolism, as nitric oxide (NO) limits T cell proliferation [5]. Inflammatory macrophages in the GI tract during acute infection and inflammation have been shown to be positive for this immune mediator. In the spleen, during *Listeria monocytogenes* infection, iNOS^+^ macrophages suppress antigen-specific T cell responses [90]. Given the prevalence of iNOS^+^ macrophages in the GI tract during inflammation, these data imply that these cells may well be capable of suppressing effector T cell responses in the GI mucosa. In line with this, a CX3CR1^+^CD11c^+^CD11b^+^ MNP in the GI tract has been shown to be capable of limiting CD4^+^ T cell proliferation [50]. This required cell-cell contact highlighting another mechanism that could be employed by macrophages to curtail gastrointestinal T cell responses.

### Macrophages in human gut inflammation

Mechanistic understanding of macrophages in human gut inflammation is much more limited than in animal models. One thing that is clear is that CD14^hi^ MNP accumulate in the inflamed gut in settings such as IBD [49], likely in response to elevated levels of the chemokines CCL2 and CCL4 [95]. Indeed, by radiolabelling of autologous blood monocytes, it was demonstrated that the CD14^hi^ cells arise from these circulating precursors [27]. These cells are the human equivalent of the Ly6C^hi^ populations in mice that become dominant during induced colitis and oral *T. gondii* infection [4, 17, 25, 104, 119]. As with recruited macrophages in the mouse, the CD14^hi^ cells in the inflamed human intestine produce high levels of TNF-α, IL-1β and IL-6 [81] and have ongoing responsiveness to microbe-derived factors [49, 105].

In addition to cytokine release, it has also been reported that macrophages in the inflamed mucosa have increased expression of CD40 that may support local interactions with pathology-driving effector T cells [7], again mirroring data from murine models [119]. Given the currently sparse data on human intestinal macrophage function, but their high relevance to disease pathology, this will no doubt be an area for tremendous future scientific study. The already strong similarities between patient samples and murine models suggest that integration of these two investigative strategies may be key to rapidly progressing our knowledge of this area.

### Long-range instruction of inflammatory gut macrophage function during infection

At steady state, much research focus has been placed on the cues present in the gut environment that locally instruct resident gut macrophage differentiation. Based on this model, during gut inflammation, factors at the affected tissue site to which infection-elicited macrophages are exposed have been of primary interest. However, a number of recent findings, including those from our group, suggest that a more holistic approach to understanding macrophage differentiation during inflammation needs to be employed.

It is well established that when stressed, tissues such as the gut can send signals to the bone marrow (BM) niche and blood to improve supply of required circulating immune populations [28, 89]. We found that during gut infection, these long-range signals can also instruct for altered functional potential. As early as 4 days after oral infection with *T. gondii*, Ly6C^hi^ monocytes developing in the BM niche acquired a characteristic CX3CR1^low^MHCII^hi^Sca-1^hi^ phenotype, which was also observed in inflammatory gut macrophages [1] suggesting that instruction of eventual macrophage differentiation may be beginning in the BM. Most importantly, when highly pure BM Ly6C^hi^ monocytes were isolated by FACS from naïve and *T. gondii*-infected animals and exposed to commensal signals they ultimately would experience in the inflamed gut, they already had enhanced capacity to produce PGE_2_ prior to BM efflux. As discussed already, we have established PGE_2_ as a critical recruited gut macrophage-derived factor regulating neutrophil-mediated immunopathology during oral *T. gondii* infection [25]. Thus, during gut infection, functional priming of inflammatory macrophages can begin in the BM.

Although PGE_2_ production was highlighted, the Ly6C^hi^ monocytes had profound transcriptional changes and also had enhanced capacity to produce anti-inflammatory IL-10 in response to bacterial ligands. These transcriptional changes were at least initiated in the direct proliferative precursor to monocytes, the common monocyte progenitor (cMoP) [36], but earlier progenitors such as the MDP were not investigated due to issues of identification in infection [1].

The precise mechanisms that the gut can use to communicate to the BM niche are not understood. During *T. gondii* infection, we identified a whole organism signalling loop in which a specific subset of gut DC (Batf3^+^) released the cytokine IL-12 in the serum that was detected by a subset of mature natural killer (NK) cells present in the BM. These NK cells produced IFN-γ locally in the BM in response to the IL-12 signal, which was critical in generating the high PGE_2_-producing CX3CR1^low^MHCII^hi^Sca-1^hi^ monocytes. Although IFN-γ was a dominant signal, whether additional signals in the BM environment are also altered following *T. gondii* infection and whether these have specific functional effects on developing monocytes and eventually their gut macrophage progeny remain unknown. One possibility is food-derived ligands such as short-chain fatty acids (SCFAs), as these have previously been shown to modify DC function prior to exit from the BM in an asthma model [106].

Intriguingly, during *T. gondii* infection, our data suggested that perturbations to monocyte-macrophage differentiation capacity might be initiated systemically, as there were dramatic increases in circulating Ly6C^hi^ monocytes with a concurrent loss of Ly6C^low^ patrolling monocytes (which are suggested to derive from the Ly6C^hi^ population [116]). This bears striking similarity to the proposed block in differentiation of monocytes to resident gut macrophages in colitis [4, 119]. The cytokine IFN-γ was implicated in this process [1], but further work will be needed to confirm whether this is due to an arrest in normal differentiation pathways (rather than, for example, increased BM output of Ly6C^hi^ monocytes) and whether these Ly6C^hi^ monocytes are also unable to differentiate into resident macrophages upon entry into the gut.

### Resident and inflammatory macrophages in resolution of inflammation

Once the infection, or other inflammation-driving factor, has been cleared or becomes tolerated in the gut, there must be a resolution phase to restore homeostasis. If this does not happen, then chronic inflammation may develop.

As occurs during inflammation, this resolution phase is also associated with striking alterations to gut macrophage subsets. One example of this is that following resolution of DSS colitis in mice, the augmented CX3CR1^int^ MNP subset returns to baseline levels [119]. Precise reasons for the loss of the CX3CR1^int^ subset are not known, but apoptosis and uptake by resident macrophages seem probable [22]. An alternative possibility, based on the idea that CX3CR1^int^ cells are blocked in their differentiation to resident gut macrophages during inflammation, is that due to alterations in the cytokine milieu, this limitation is removed. As a result, many of the CX3CR1^int^ cells would “disappear” from the gut by differentiating into resident gut macrophages.

Whatever the case, it seems that gut monocytes/macrophages are important in gut repair and resolution of inflammation. In particular, there is a delay in DSS colitis resolution in animals that lack TGF-β signalling on monocytes/macrophages (CD68-dnTGFβRII) [78], while deletion of MyD88 signalling in myeloid cells limits gut healing [62].

Although not well studied specifically in the gut, one set of factors that are important in restoration of homeostasis in all tissues are pro-resolving lipid mediators [91]. These factors, as well as limiting influx of additional inflammatory cells, can promote the uptake of apoptotic cells by macrophages [20, 29]. Types of lipid mediators include lipoxins, resolvins and protectins that can be produced by multiple cell types including macrophages [91]. One factor that has been demonstrated to favour production of lipoxins is prostaglandins, in particular PGE_2_. For example, in resolving inflammatory exudates, it has been suggested that PGE_2_ and PGD_2_ can stimulate production of a functional enzyme for lipoxin in neutrophils [57]. Based on our studies in *T. gondii* infection, this raises the intriguing possibility that Ly6C^hi^ monocytes/macrophages not only limit neutrophil activation but also deviate their function towards a lipoxin-producing pro-resolution state. Relating to this, macrophages may also be able to regulate wound healing directly by interacting with epithelial progenitor cells during colonic wound healing [77]. Again, lipid mediators may play a role in this process as COX-2 expression, a critical factor in PGE_2_ production, has been linked to protection of progenitor populations at other tissue sites during inflammation [60].

As well as repairing the barrier, another feature of return to homeostasis is the restoration of the adaptive immune compartment, which can become dramatically perturbed during an inflammatory response [73]. At this time, it is unclear how the mononuclear phagocytes of the gut may act together to support this return, but given their suggested role in expanding Tregs at steady state, it is likely that gut-resident macrophages are important to this process.

With the recent advances in technologies for the generation of transgenic murine systems, novel tools for the temporal knockout of specific genes in defined macrophage/monocyte subsets are likely to become increasingly available. This will allow candidate factors identified as being important to resolution to be depleted during this phase of the inflammatory response without impairing initiation of disease. Already, the tamoxifen-inducible Cx3CR1^creER^ mouse that knocks out factors in CX3CR1^hi^-resident gut macrophages holds much promise for this purpose [116].

### Concluding remarks

Utilising novel transgenic animals alongside cutting-edge cytometric and genomic approaches, recent years have seen an explosion in our understanding of macrophage biology in general and more specifically in the gut. In particular, the field is beginning to better define the diverse functions that these cells play in tissue homeostasis and how they can be manipulated in disease states.

With the advent of single-cell RNA sequencing, it will become more straightforward to define functionally distinct macrophage subsets within complex and potentially inflamed tissue environments. One aspect of macrophage biology that has been largely overlooked in recent years is precise localisation within the tissue. For example, in the gastrointestinal tract, there are diverse structural components to the tissue, e.g. muscle, nerves, blood vessels and epithelium. How macrophage function might be tailored to each of these niches is only just beginning to be understood.

Another area in the functional diversity arena is to further understand the differential roles of resident macrophages versus inflammatory macrophages in disease states. It is clear that resident macrophages are often maintained in the inflammatory environment but are not acquiring classical activation markers. Whether these cells play a role during the active inflammatory event or in restoring homeostasis post challenge is extremely unclear. This is critical to understand as it would help elucidate specific macrophage factors that can be manipulated at defined time points in the inflammation resolution cycle to alter outcome.

Whatever the future of macrophage research holds, given the importance of intestinal macrophages to the maintenance of homeostasis and disease progression, better understanding the development and functions of this cell type will no doubt yield novel strategies that can inform development of therapies to improve patient outcome in inflammatory diseases such as IBD.
